# Cesarean Section in a Morbidly Obese Patient With Severe Preeclampsia and Pulmonary Edema: A Case Report

**DOI:** 10.7759/cureus.65877

**Published:** 2024-07-31

**Authors:** Abduljaleel Ethy Ahammedunni, Nadine B Nour, Kundan Das

**Affiliations:** 1 Department of Anaesthesia and Intensive Care, Latifa Hospital, Dubai Health, Dubai, ARE

**Keywords:** spinal anesthesia, morbid obesity, pulmonary edema, preeclampsia, cesarean

## Abstract

Severe preeclampsia and pulmonary edema pose significant challenges for an anesthesiologist. Pregnancy is associated with major physiologic changes to meet the increased demands of the mother and fetus. Preeclampsia complicates this balance by adding additional stress to the mother and baby. Pulmonary edema is a rare complication of preeclampsia, and it is a potentially life-threatening condition. Meticulous care is needed in the anesthetic management of this condition, especially when the patient is morbidly obese and presents for an emergency cesarean section.

## Introduction

Preeclampsia is characterized by hypertension and proteinuria after 20 weeks of gestation [1,2]. Cardiorespiratory, renal, hepatic, cerebral, and hematological involvement can occur. The condition can also affect the well-being of the baby. One rare complication of severe preeclampsia is pulmonary edema, which is life-threatening. We are reporting a case of pulmonary edema resulting from severe preeclampsia in a morbidly obese patient. The anesthetic management of the cesarean section in this patient with morbid obesity is discussed.

## Case presentation

A 34-year-old pregnant lady presented in the emergency department with a two-day history of cough, breathlessness, and reduced fetal movements. She had a previous cesarean section five years back. Her gestational age was 38 weeks, and she had irregular follow-ups during pregnancy. She had missed an appointment for a cesarean section the previous week. She was known to have essential hypertension and gestational diabetes mellitus, for which she was taking labetalol and Glucophage. She was morbidly obese, with a weight of 125 kg and a BMI of 54.1. Both lower limbs, the abdominal wall, and the face were edematous. Oxygen saturation was 93-94% in room air. Her blood pressure was 163/102 mmHg, but she gave no history of headache, epigastric pain, or blurring of vision. Non-invasive blood pressure monitoring was difficult due to morbid obesity. Tendon reflexes were normal. The heart rate was 94/minute, and the respiratory rate was 32-35/minute. The fetal heart rate was 144/minute. Bilateral, coarse crepitations were present on chest auscultation.

**Figure 1:**
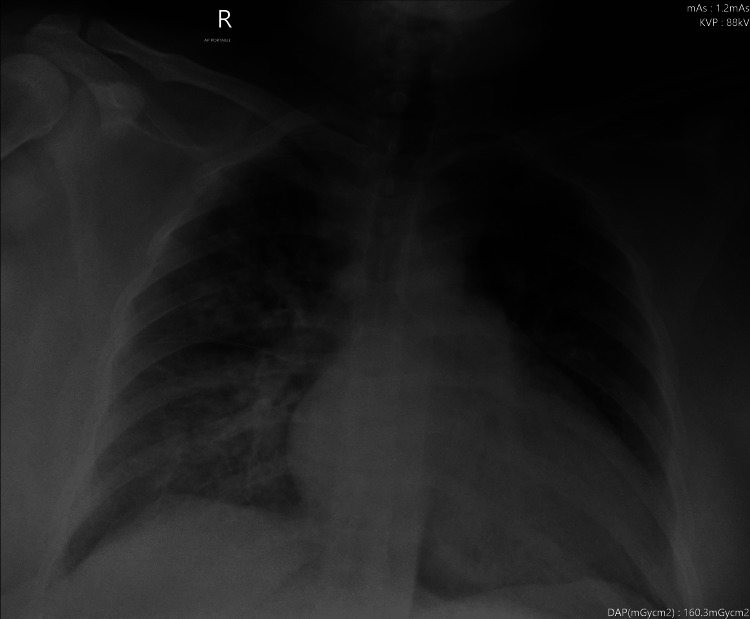
Chest X-ray on admission showing pulmonary edema

High-concentration oxygen was started at 15 liters/minute through a face mask, and she was shifted to the ICU for stabilization before the cesarean section. Furosemide injection 80 mg IV and labetalol 20 mg IV were given. A radial artery cannula was inserted to monitor the blood pressure. Lung ultrasound showed B lines bilaterally. Blood samples were sent for CBC, coagulation profile, and renal and liver function tests. Invasive blood pressure was 220/120 mmHg. A magnesium sulfate 4 gm intravenous bolus was given. After the blood gas results, she was put on a high-flow nasal cannula of oxygen at 40 L/minute with a FiO2 of 0.5 in a propped-up position. Foley's catheter drained 30 mL of urine. The chest X-ray showed signs of pulmonary edema (Figure 1). Her ECG was normal. A bedside echocardiogram showed preserved left ventricular function. Her blood pressure was controlled with a labetalol infusion, and a magnesium sulfate infusion was started. She was put on an insulin sliding scale to control her blood sugar.

Her hemoglobin was 8.1 g/dL, and troponin T was 15 ng/L (Table 1). Liver function and coagulation tests were normal. The urine albumin-creatinine ratio was 151.2. The obstetrician decided to transfuse two units of packed red cells. She was monitored in the ICU with a plan for an emergency cesarean if there was deterioration in her maternal or fetal condition. Her breathing improved gradually, and her respiratory rate improved to 24/minute in four hours. She was put on oxygen through a nasal cannula at 4 L/minute. Blood pressure was 152/82 mmHg. Serial arterial blood gas results are given in Table 2.

**Table 1:** Lab results during the hospital stay WBC: white blood cell, eGFR: estimated glomerular filtration rate, NT-proBNP: N-terminal pro-B-type natriuretic peptide

Component	Reference range	On admission	Before cesarean	After cesarean	Day 2	Day 3
WBC	3.6-11x10^9^/L	10.1	10.8	10.1	-	8.3
Hemoglobin	12-15g/dL	8.2	10.4	8.5	-	9.2
Hematocrit	%	26.8	33.3	27.9	-	29.5
Platelets	150-410x10^9^/L	357	304	325	-	337
Serum sodium	136-145 mmol/L	137	137	137	137	-
Serum potassium	3.3-4.5 mmol/L	3.9	3.4	3.7	4.1	-
Serum bicarbonate	20-28 mmol/L	17.4	17.9	18.5	24.5	-
Urea	12-40 mg/dL	29	25	27	25	-
Serum creatinine	0.5-0.9 mg/dL	0.57	-	0.7	-	-
eGFR	>60 ml/min/1.73 m^2^	122.2	-	116.3	-	-
NT-proBNP	<125 pg/ml	-	610		-	-
Blood sugar	60-120 mg/dL	85	81	106	115	129

**Table 2:** Serial blood gas results during the first 24 hours of ICU stay ICU: intensive care unit

Component	Reference range	Sample 1	Sample 2	Sample 3	Sample 4
pH	7.35-7.45	7.427	7.379	7.349	7.377
pCO2	35-45 mmHg	27.1	32.0	35.9	36.0
pO2	83-108 mmHg	121	120	64.2	93.2
Bicarbonate	21-28 mmol/L	19.7	19.8	20.0	21.4
Hemoglobin	11.0-15.0 g/dL	8.1	8.5	10.9	10.7
Sodium	134-143 mmol/L	140	141	141	141
Potassium	3.4-5.0 mmol/L	3.7	3.2	3.8	3.7
Chloride	97-108 mmol/L	113	111	111	109
Ionized calcium	1.15-1.29 mmol/L	1.13	1.13	1.08	1.14
Lactate	0.5-1.6 mmol/L	0.6	1.0	1.3	1.1
Base deficit	mmol/L	6.5	6.2	5.9	4.1

She was shifted for a cesarean section seven hours after the presentation. A cesarean section was done under spinal anesthesia. Lumbar puncture was done in a sitting position in L4/5 intervertebral space. Hyperbaric bupivacaine 11 mg, fentanyl 10 mcg, and morphine 100 mcg were given intrathecally. The patient was positioned in a ramped-up position with a left uterine tilt. The level of spinal anesthesia was confirmed at T4 before starting surgery. She received oxygen through a nasal cannula at 4 L/mt. Carbetocin 100 mcg IV was given after the delivery of the baby for uterine contractions. The intraoperative period was uneventful, and she had stable blood pressure during surgery. She received an additional one unit of packed cells and two units of fresh frozen plasma during surgery. Blood loss was 600 ml, and 150 ml of urine was drained during surgery. The surgery lasted one hour and 50 minutes. She was shifted to the ICU for observation. The magnesium sulfate infusion was continued for 24 hours. She had an uneventful postoperative period, and blood pressure was controlled with oral labetalol. She was discharged on the fifth postoperative day.

## Discussion

Preeclampsia affects 5% to 7% of all pregnancies and causes more than 70,000 maternal deaths and over 500,000 fetal deaths worldwide annually [2]. Abnormal placentation is thought to be the underlying pathology. Left ventricular systolic and diastolic dysfunction can co-exist. Airway edema, reduced glomerular filtration, thrombocytopenia, liver dysfunction, and deranged coagulation are possible associations. Many of these patients have other comorbidities like anemia, gestational diabetes mellitus, obesity, and chronic hypertension. The incidence of maternal cardiovascular events during hospitalization for delivery is double in preeclampsia and five times in severe preeclampsia compared to those without preeclampsia [3]. Preeclampsia also predisposes to long-term cardiovascular risks, which include coronary heart disease, cerebrovascular disease, and heart failure [4,5]. Once confirmed, these patients require regular follow-up and control of their blood pressure. The goal is to maintain normal cardiac output and uteroplacental blood flow and prevent the development of seizures and strokes. Pulmonary edema has an incidence of 2.9% in patients with severe preeclampsia [6]. Various other causes of pulmonary edema during pregnancy should be excluded. This includes pre-existing or pregnancy-related cardiac diseases and pulmonary edema related to fluid overload or drug usage [7].

Cough, breathlessness, orthopnea, and agitation are usual symptoms of pulmonary edema, and the patient may exhibit tachypnea, tachycardia, crepitations on chest auscultation, and reduced oxygen saturation. Chest X-rays and bedside ultrasounds help confirm pulmonary edema. Patients should receive oxygen through a non-invasive continuous positive airway pressure device or high-flow nasal cannula. Intravenous frusemide and a bolus of magnesium sulfate are usually used in management, along with control of blood pressure [8,9]. Intravenous fluids generally should be restricted to less than 80 ml/hour in patients with preeclampsia, as they are prone to fluid overload [8,10]. Magnesium produces systemic, cerebral, and uterine vasodilatation [11]. Intravenous labetalol, nitroglycerine, or hydralazine can be used if blood pressure is high.

Morbid obesity is another important factor complicating the management of these patients. Obesity predisposes to other comorbidities like hypertension and gestational diabetes mellitus [12,13]. They often suffer from sleep-disordered breathing. Obese pregnant patients have a 15-20% incidence of obstructive sleep apnea [13,14]. They usually have mucosal hyperemia, narrowing of oropharyngeal diameter, a high Mallapatti score, increased oxygen consumption, and reduced functional residual capacity of the lung. Intermittent hypoxia leads to endothelial dysfunction and pulmonary hypertension. A careful evaluation of the airway is necessary, including a history and examination, to rule out obstructive sleep apnea. Snoring at sleep, disturbed sleep, and daytime sleepiness are indicative of sleep apnea. A neck circumference of more than 40 cm and a STOPBANG score of more than 3 are significant findings. General anesthesia is associated with significant complications in morbidly obese patients, usually due to a difficult airway. The use of regional anesthesia in the obese reduces the risks of difficult intubation and acid aspiration and also provides safer and more effective postoperative analgesia [14,15]. However, spinal anesthesia is technically difficult for obese patients, and longer spinal needles may be required. The cephalad spread of spinal anesthetics can impair breathing.

Spinal anesthesia is considered safer in patients with severe preeclampsia [16]. Spinal anesthesia was rarely used in the past for fear of maternal hypotension and reduced uteroplacental blood flow. However, it has been shown that the incidence and severity of hypotension are lower in preeclampsia compared to those without preeclampsia. The requirement of ephedrine to treat hypotension is also less in preeclampsia [17,18]. Also, there is no difference in the APGAR score of the baby or umbilical artery pH. General anesthesia is associated with airway complications, pulmonary aspiration, and the risk of stroke. For these reasons, neuraxial anesthesia is now recommended for a cesarean section in preeclampsia if there is no epidural catheter already in place. There is no significant difference between spinal and epidural anesthesia, as hypotension is easily treatable in both. A recent platelet count of more than 80,000 is considered safe for neuraxial techniques. A coagulation test is advisable as part of the liver function test if the platelet count is less than 150,000 [16].

## Conclusions

This case report discusses the anesthetic management of a cesarean section in a patient with a BMI of 52.1 who presented with pulmonary edema resulting from severe preeclampsia. A thorough understanding of the pathophysiology and clinical course of preeclampsia is necessary for the successful management of these patients. Prompt management of pulmonary edema and control of blood pressure is needed before proceeding to a cesarean section whenever possible. Regional anesthesia has definite advantages over general anesthesia, as the latter is associated with potentially serious complications. Spinal anesthesia, compared to general anesthesia, also helps to avoid many complications related to morbid obesity.
